# Supplementary material for: Dynamic control of decision and movement speed in the human basal ganglia
Source: Nat Commun. 2022 Dec 7;13:7530. doi: 10.1038/s41467-022-35121-8 (PMC9729212; doi:10.1038/s41467-022-35121-8)
Supplement: Supplementary file 1 — Supplementary Information [file 41467_2022_35121_MOESM1_ESM.pdf]

Dynamic control of decision and movement speed in the human basal ganglia

Supplementary information:

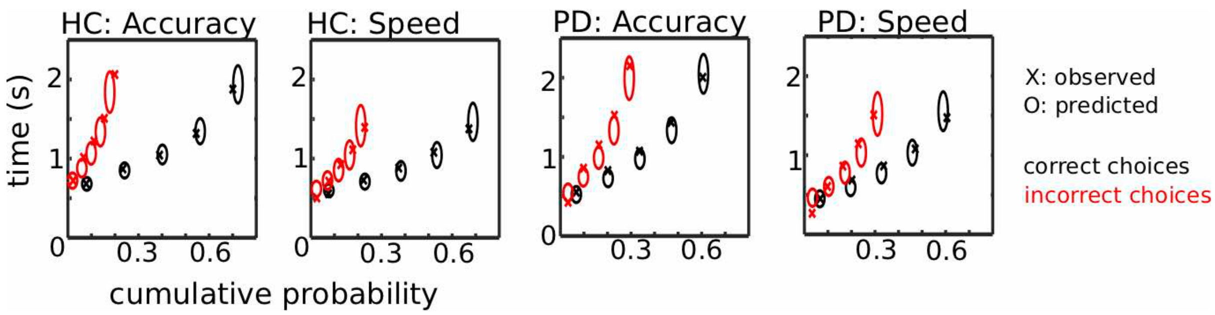

Supplementary figure 1. Quantile probability plots showing the observed (indicated by a cross) and predicted (indicated by ellipses) cumulative probabilities of reaction times separately for the two groups (Parkinson's disease (PD) & healthy controls (HC)) and Instructions (speed & accuracy). Correct choices are shown in black, incorrect choices in red. The width of the circles reflects prediction uncertainty (standard deviation of the posterior predictive distribution).

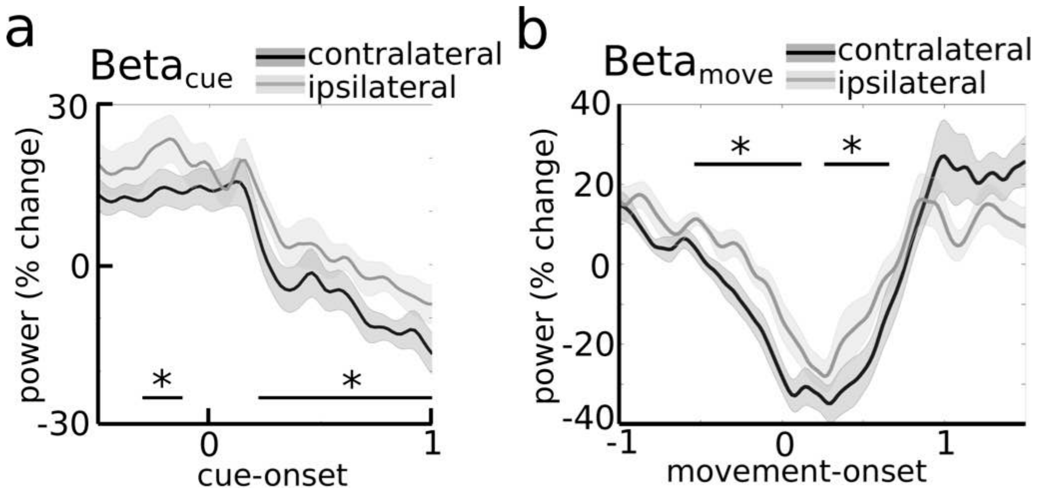

Supplementary figure 2. Lateralization of subthalamic beta power. **A.** Changes in beta power aligned to the cue averaged across participants (n=13). **B.** Changes in beta power aligned to the movement averaged across participants (n=13). Black bars with a * indicate time windows where beta power was lower in contralateral (black) compared to ipsilateral (grey) subthalamic nucleus ($P_{cluster} < 0.05$, corrected for multiple comparisons using cluster-based permutation tests and two-sided alpha-level of 0.05). Comparing $Beta_{move}$ and $Beta_{cue}$ (see text) between hemispheres showed that $Beta_{move}$ was significantly lower contralateral vs. ipsilateral (CI [-0.168:-0.024], P = 0.014, t-test with two-sided alpha-level of 0.05) while there was no significant difference in $Beta_{cue}$ (CI [-0.064:+0.061], P = 0.959, t-test with two-sided alpha-level of 0.05). Shaded areas represent SEM.

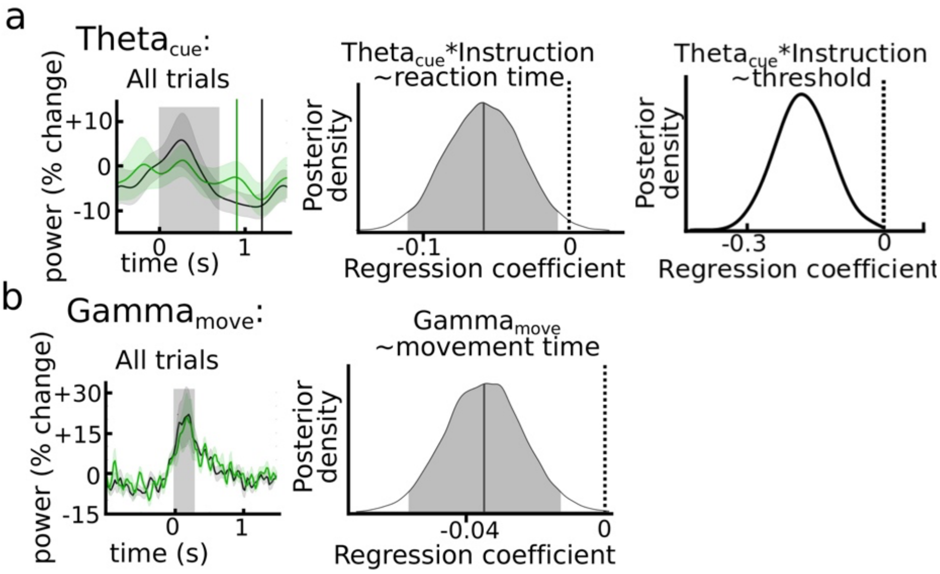

Supplementary figure 3. Changes in subthalamic theta and gamma power. **A.** Results for cue-aligned changes (0-700 ms postcue) in theta (4-8 Hz) power (Theta$_{cue}$) averaged across participants (n=13). Across trials Theta$_{cue}$ did not differ after speed (green) vs. accuracy (black) instructions (CI [-0.093:+0.054], P=0.571, t-test with two-sided alpha-level of 0.05, left panel), but its trial-by-trial relationship with reaction times depended on Instruction (CrI of IA [-0.107:-0.004], middle panel). In line with the behavioural results, HDDM showed a significant interaction of Theta$_{cue}$ and Instruction on decision thresholds (CrI of IA [-0.291:-0.063], CrI of main effect [-0.009:+0.172], shown in right panel, but not on drift rate (CrI [-0.159:+0.078]) or non-decision times (CrI [-0.016:+0.012]). See supplementary table 2 for further statistical tests of Theta$_{cue}$ on movement and reaction times **B.** Results for movement-related (0-300 ms post-movement) changes in gamma (55-80 Hz) power (Gamma$_{move}$) averaged across participants (n=13). Gamma power showed a strong increase during the movement, but this increase was not different between speed (green) and accuracy (black) instructions (CI [-0.065:+0.014], P=0.187, t-test with two-sided alpha-level of 0.05, left panel). It showed a significant single-trial relationship with movement times irrespective of instruction (CrI [-0.058:-0.013], right panel), i.e. higher gamma power predicted faster movements. See supplementary table 2 for further statistical tests of Gamma$_{move}$ on movement and reaction times. Shaded areas around % power change represent SEM and grey vertical boxed areas in the left panels indicate time windows from which power was extracted. Vertical green and black lines in the left panel of panel A indicate mean reaction time for, respectively, speed and accuracy instructions.

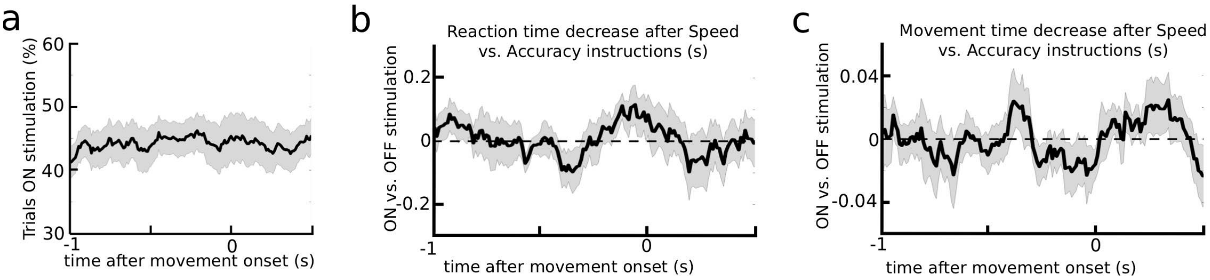

Supplementary figure 4. Behavioral effects of bilateral burst stimulation aligned to movement onset. **A.** Bursts were given at random time points throughout the experiment resulting in stimulation occurring on ~50% of trials for any given 100ms moving time window across participants (n=10). **B.** Stimulation had no significant effect on patients' ability to adjust reaction times across participants (n=10, corrected for multiple comparisons using cluster-based permutation tests and two-sided alpha-level of 0.05). **C.** Stimulation had no significant effect on patients' ability to adjust movement times across participants (n=10, corrected for multiple comparisons using cluster-based permutation tests and two-sided alpha-level of 0.05). Shaded areas represent SEM.

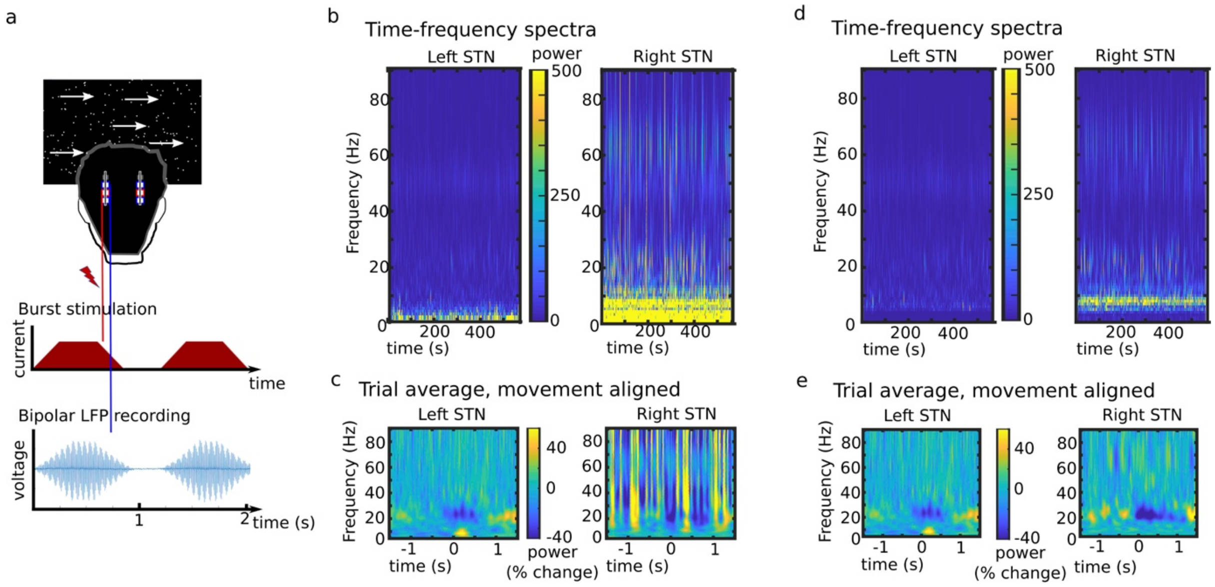

Supplementary figure 5. Stimulation-induced artefacts. **A**. Bursts of stimulation led to clearly visible artefacts in the un-preprocessed local field potentials (lower panel). **B&C**. Raw (B) and average (C) time-frequency spectra without artifact correction (see methods for details regarding artifact correction) for a representative patient. The spectral properties of stimulation-related artefacts were not restricted to the stimulation frequency and its harmonics and, when strongly expressed (here right STN), obliterated the normal movement-related beta power modulation. When the artifact was less pronounced normal beta modulation was present even without artifact correction (here left STN). **D&E**. Time-frequency spectra after artifact correction for the same patient as in B&C. Beta modulation is now visible also in the right STN.

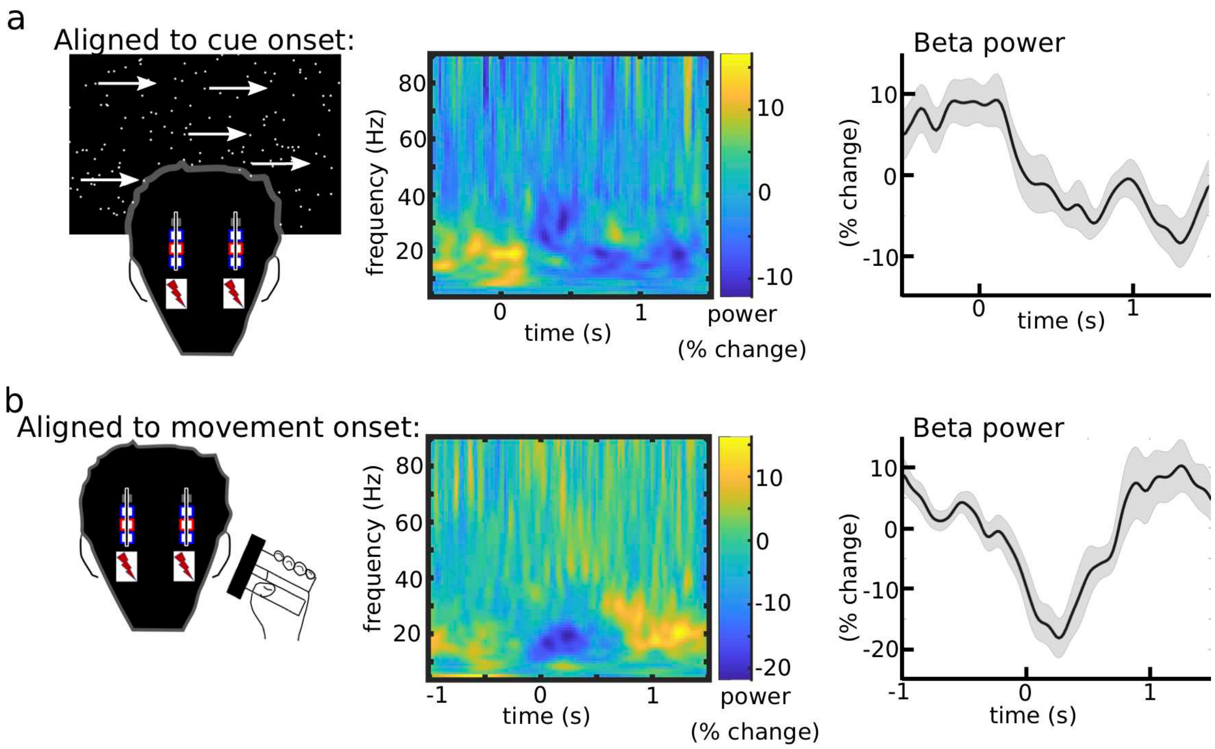

Supplementary figure 6. Recovery of beta power modulation during stimulation trials. **A.** Grand average of all trials from the stimulation session aligned to the moving dots cue for all frequencies (left) and mean beta power (right) averaged across participants (n=10) demonstrating the cue-related reduction in beta power, which was also observed in the off stimulation session. **B.** Grand average of all trials from the stimulation session aligned to movement onset for all frequencies (left) and mean beta power (right) averaged across participants (n=10) demonstrating the movement-related reduction in beta power, which was observed in the off stimulation session. Shaded areas in the right panels represent SEM.

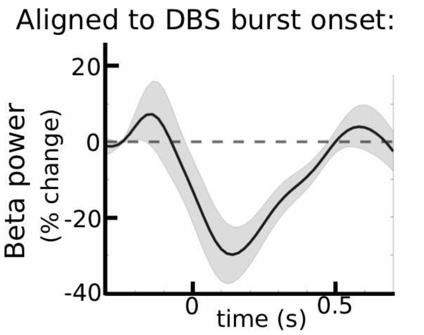

Supplementary figure 7. Effect of stimulation bursts (mean duration: 250 ms +/- 100) on beta power averaged across participants (n=10). Beta power is aligned to onset of stimulation (after ramping) and normalized to the time period where no stimulation was applied. Stimulation led to a ~30% decrease in beta power, which returned to baseline after ~0.5 s. Shaded areas represent SEM.

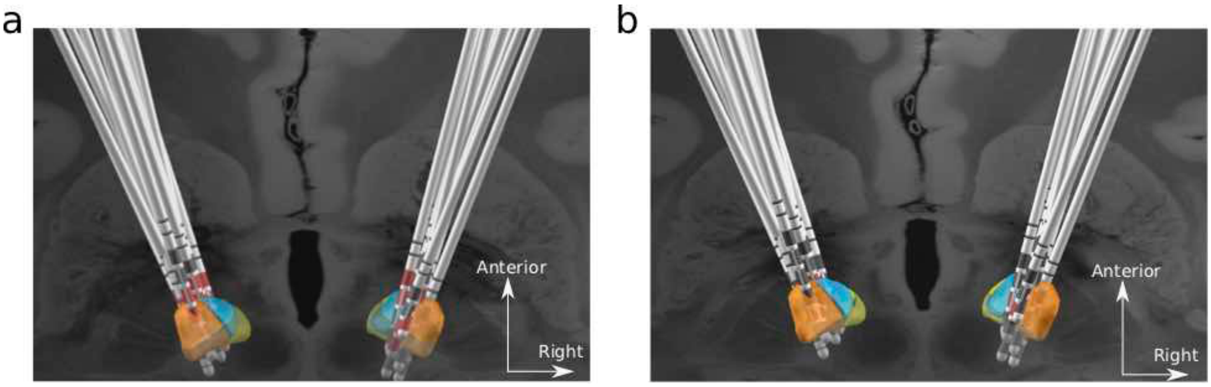

Supplementary figure 8. Electrode localization. **A.** Reconstructed bilateral leads are overlaid on subthalamic nucleus (STN; orange: STN area mainly connected to motor regions, blue: STN area mainly connected to associative regions, yellow: STN area mainly connected to limbic regions) for all patients where pre- and postoperative imaging was available (n=10). Electrodes from which bipolar local field potential signals were analyzed are marked in red. **B.** Same as A, but marked electrodes indicate contacts which were used for burst stimulation.

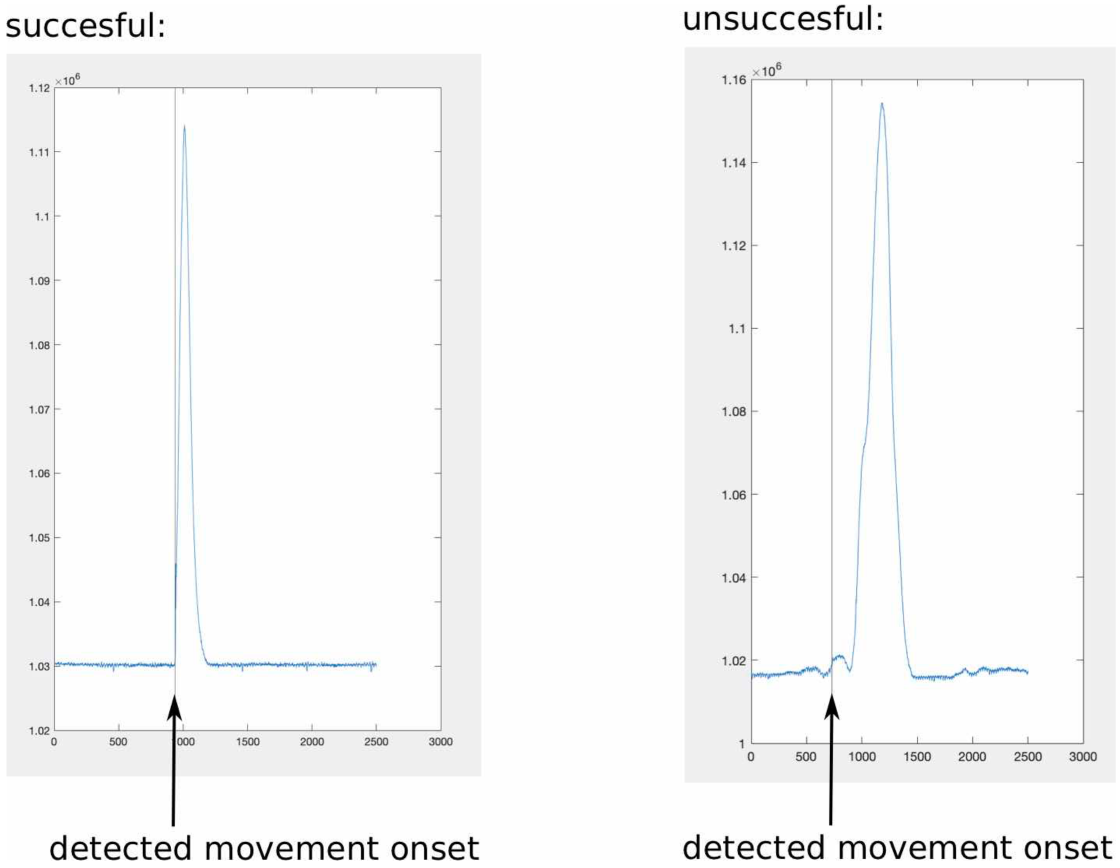

Supplementary figure 9. X-axis shows time in ms (1000 ms corresponds to the force surpassing the pre-defined threshold), y-axis is force (au). The arrow and vertical line indicate movement onset as detected by the automatic algorithm (see methods), which in the right panel was triggered by a small variation in force, presumably due to subtle action tremor. Therefore, in a significant proportion of trials it was necessary to set the onset manually. To avoid any putative group bias (manual adjustments are mainly necessary in patients, while the data from healthy people usually does not require this) we opted to always set the onset manually blinded to trial-type.

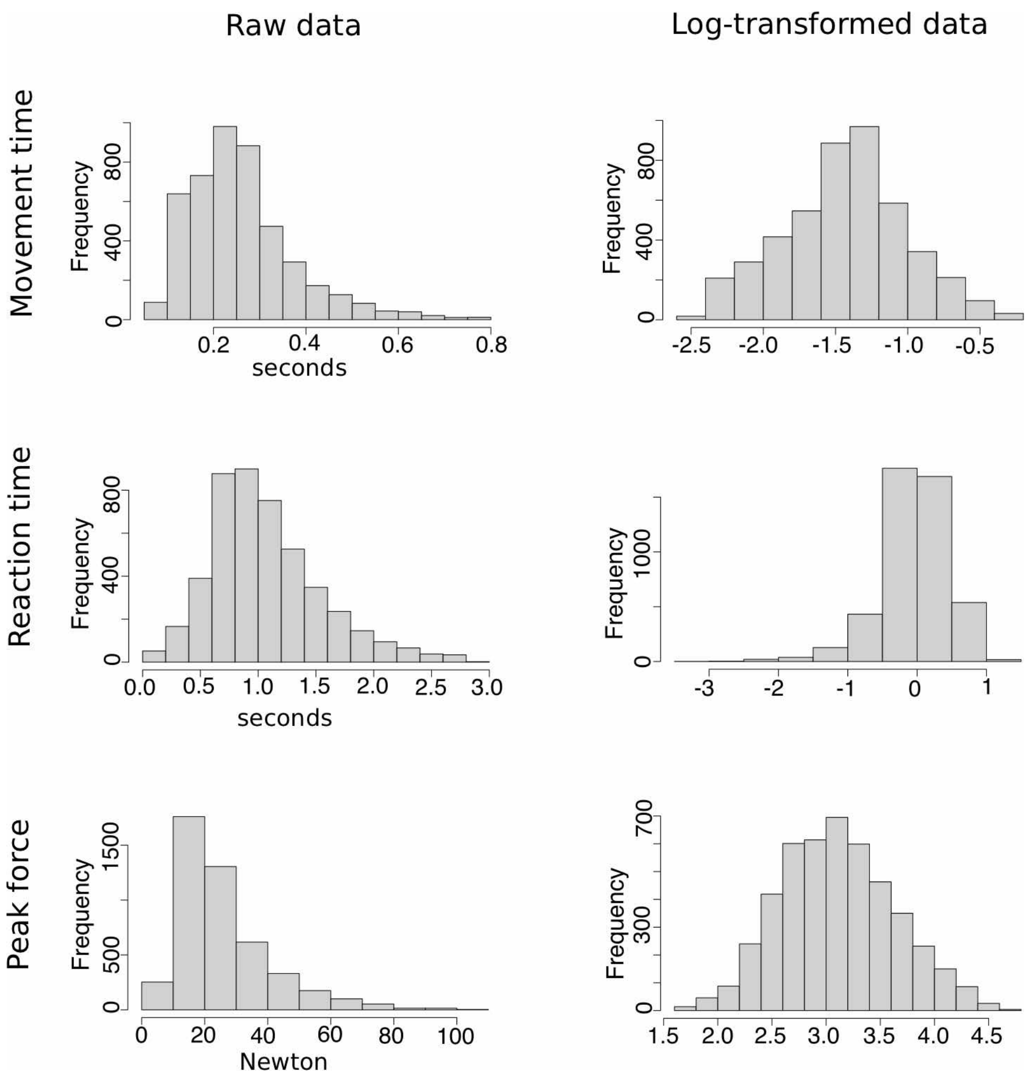

Supplementary figure 10: Distribution of single trial raw (left column) and log-transformed (right column) behavioral data. Raw movement times, reaction times and peak force had a heavy tail. Since the Bayesian model assumed a normal distribution this data was log transformed, but the significant key results did not change when omitting this step (listed in supplementary table 4).

#	Age & gender	UPDRS-III OFF/ON levodopa	UPDRS-III limb OFF/ON DBS	Disease duration	Main symptom	Reason for surgery	Medication (LEDD)	DBS site and lead	DBS parameters Left / Right
1	male	49 / 18	n/a	12	Tremor	Tremor	1881 mg	London, Boston Scientific DB 2202™	n/a
2	male	n/a	n/a	4	Tremor	Tremor	533 mg	London, Medtronic 3389™	n/a
3	male	33 / 14	22 / 17	6	Bradykinesia	ON-OFF fluctuations	1569 mg	Mainz, Abbott 6170™	D-D, 1.7 mA, 0.215 s
4	female	25 / 17	24 / 20	7	Bradykinesia	Wearing OFF	1285 mg	Mainz, Abbott 6170™	D-V, 2.4 mA, 0.184 s
5	male	32 / 24	10 / 4	5	Bradykinesia and tremor	Dyskinesia	400 mg	Mainz, Abbott 6170™	D-D, 3.0 mA, 0.230 s
6	male	28 / 12	n/a	16	Bradykinesia	ON-OFF fluctuations	1170 mg	Mainz, Abbott 6170™	n/a
7	male	59 / 50	n/a	13	Bradykinesia	ON-OFF fluctuations	1448 mg	Mainz, Abbott 6170™	n/a
8	male	46 / 31	23 / 13	13	Bradykinesia	Dyskinesia	1548 mg	Mainz, Abbott 6170™	V-V, 1.3 mA, 0.2 s
9	male	n/a	19 / 13	19	Tremor	Wearing OFF	1480 mg	Mainz, Abbott 6170™	D-D, 2.5 mA, 0.192 s
10	male	44 / 20	23 / 13	16	Bradykinesia	ON-OFF fluctuations	1000 mg	Mainz, Abbott 6170™	D-D, 2.5 mA, 0.192 s
11	male	34 / 32	27 / 21	4	Tremor	Tremor	450 mg	Mainz, Abbott 6170™	D-D, 2.0 mA, 0.153 s
12	male	63 / 33	37 / 31	30	Bradykinesia	ON-OFF fluctuations	892 mg	Mainz, Abbott 6170™	D-D, 1.1 mA, 0.169 s
13	female	45 / 26	n/a	11	Tremor	Tremor	900 mg	Mainz, Abbott 6170™	n/a
14	male	42 / 23	32 / 26	6	Bradykinesia	Gait difficulties	1514 mg	Mainz, Abbott 6170™	D-D, 2.5 mA, 0.192 s
15	male	24 / 9	36 / 20	10	Bradykinesia	Dyskinesia	455 mg	Mainz, Abbott 6170™	D-D, 2.0 mA, 0.153 s

Supplementary table 1. Disease duration are given in years. Clinical scores are given as total score of the MDS Unified Parkinson's disease rating scale (UPDRS) part III for levodopa ON/OFF and as items 3-8 & 14-18 (limb scores) for DBS ON/OFF. Medication is given in levodopa-equivalent daily dose (LEDD) [1, 2]. All patients received levodopa, 12 patients received a dopamine-agonist, 10 patients a catechol-O-methyltransferase (COMT) inhibitor, 8 patients a monoamine-oxidase (MAO) inhibitor and 3 patients amantadine. D and V indicate whether the respectively more dorsal or ventral contact was chosen as active contact in the left and right hemisphere, after which DBS intensity is given in mA and ramp time is given in seconds. n/a, not available. Individual age is not given to limit patient identifying information. The age range of patients was 49 to 79 years.

Statistical test	CrI (Bayesian)	CI, t- & p-value (Frequentist)
<u>Reaction time:</u>		
Instruction	**-0.429 : -0.139**	**-0.421:-0.149, t=-4.10, p<0.001**
Group	-0.101 : +0.249	-0.089 : +0.249, t=0.93,p=0.362
Instruction*Group	-0.152 : +0.235	-0.142 : +0.227, t=0.45,p=0.656
<u>Movement time:</u>		
Instruction	**-0.126 : -0.043**	**-0.125 : -0.046, t=-4.22, p<0.001**
Group	**-0.717 : -0.189**	**-0.718 : -0.202, t=-3.49, p=0.002**
Instruction*Group	-0.011 : +0.100	-0.008 : +0.098, t=1.66, p=0.110
<u>Accuracy rates:</u>		
Instruction	-0.349 : +0.176	-0.336 : +0.171, z=-0.67,p=0.505
Group	**+0.068 : +1.051**	**+0.134 : +1.073, z=2.61,p=0.009**
Instruction*Group	-0.411 : + 0.299	-0.394 : +0.286, z=-0.34,p=0.736
<u>Reaction time (effect of accuracy):</u>		
Accuracy	-0.110 : +0.047	-0.110 : +0.044, t=-0.83, p=0.411
Accuracy*Instruction	**+0.042 : +0.223**	**+0.046 : +0.224, t=2.97, p=0.003**
Accuracy*Group	-0.198 : +0.018	-0.196 : +0.016, t=-1.65, p=0.107
Accuracy*Instruction*Group	-0.212 : +0.025	-0.214 : +0.021, t=-1.62, p=0.106
<u>Movement time (effect of accuracy):</u>		
Accuracy	-0.054 : +0.020	-0.051 : +0.020, t=-0.84,p=0.406
Accuracy*Instruction	-0.061 : +0.032	-0.060 : +0.031, t=-0.61,p=0.541
Accuracy*Group	-0.024 : +0.077	-0.023 : +0.074, t=1.01,p=0.318
Accuracy*Instruction*Group	-0.057 : +0.066	-0.056 : +0.064, t=0.12,p=0.909
<u>Peak Force:</u>		
Instruction	-0.061 : +0.040	-0.058 : +0.037, t=-0.43,p=0.669
Group	-0.482 : +0.237	-0.466 : +0.197, t=-0.79,p=0.436
Instruction*Group	-0.109 : +0.023	-0.106 : +0.021, t=-1.32,p=0.198
<u>Movement time (effect of response side):</u>		
Response hand	-0.069 : +0.080	-0.065 : +0.074, t=0.12,p=0.902
Response hand*Group	-0.068 : +0.134	-0.058 : +0.131, t=0.77,p=0.450
<u>Movement time (effect of trial number):</u>		
Trial number	-0.001 : +0.001	-0.001 : +0.001, t=-0.06,p=0.949
Trial number*Group	-0.001 : +0.002	-0.001 : +0.001, t=0.239,p=0.814
<u>Single trial regression movement time~reaction time:</u>		
Movement time	**-0.099 : -0.002**	**-0.096 : -0.004, t=-2.13,p=0.040**
Movement time * Group	-0.050 : +0.080	-0.046 : +0.079, t=0.51,p=0.611
Movement time * Instruction	-0.052 : +0.034	-0.054 : +0.033, t=-0.48,p=0.632
Movement time * Group * Instruction	-0.019 : +0.087	-0.018 : +0.089, t=1.30,p=0.193
<u>Effect of LFP on movement time:</u>		

Beta$_{cue}$	-0.014 : +0.027	-0.015 : +0.027, t=0.58,p=0.561
Beta$_{move}$	-0.015 : +0.026	-0.016 : +0.025, t=0.43,p=0.666
Gamma$_{move}$	**-0.058 : -0.013**	**-0.057 : -0.013, t=-3.08,p=0.002**
Beta$_{cue}$*Instruction	-0.048 : +0.014	-0.049 : +0.015, t=-1.03,p=0.303
Beta$_{move}$*Instruction	**+0.010 : +0.069**	**+0.010 : +0.070, t=2.58,p=0.010**
Gamma$_{move}$*Instruction	-0.014 : +0.051	-0.013 : +0.050, t=1.15,p=0.251
Beta$_{move}$ (Accuracy)	-0.018 : +0.025	-0.018 : +0.025, t=0.31,p=0.760
Beta$_{move}$ (Speed)	**+0.020 : +0.068**	**+0.020 : +0.067, t=3.57,p<0.001**
Theta$_{cue}$	-0.039 : +0.025	-0.038 : +0.021, t=-0.58,p=0.563
Theta$_{cue}$*Instruction	-0.088 : +0.009	-0.087 : +0.009, t=-1.58,p=0.115
Effect of LFP on reaction time:		
Beta$_{cue}$	**-0.120 : -0.033**	**-0.120 : -0.033, t=-3.46,p<0.001**
Beta$_{cue}$ (RT < 0.4 s excluded)	**-0.076 : -0.014**	**-0.076 : -0.014, t=-2.88,p<0.001**
Beta$_{move}$	-0.033 : +0.052	-0.032 : 0.052, t=0.47,p=0.640
Gamma$_{move}$	-0.001 : +0.090	-0.001 : +0.090, t=1.92,p=0.055
Beta$_{cue}$*Instruction	-0.020 : +0.110	-0.019 : +0.112, t=1.39,p=0.164
Beta$_{move}$*Instruction	-0.095 : +0.028	-0.096 : +0.028, t=-1.08,p=0.280
Gamma$_{move}$*Instruction	-0.097 : +0.033	-0.097 : +0.033, t=-0.95,p=0.340
Theta$_{cue}$	-0.001 : +0.063	-0.001 : +0.063, t=1.90,p=0.057
Theta$_{cue}$*Instruction	**-0.107 : -0.004**	**-0.107 : -0.003, t=-2.06,p=0.039**
Theta$_{cue}$ (Accuracy)	-0.007 : +0.064	-0.007 : +0.064, t=1.57,p=0.118
Theta$_{cue}$ (Speed)	-0.048 : +0.017	-0.047 : +0.017, t=-0.93,p=0.355
Relationship between Beta$_{move}$ and Beta$_{cue}$:		
Beta$_{cue}$	-0.099 : +0.032	-0.099 : +0.032, t=-1.48,p=0.139
Beta$_{cue}$ * Instruction	-0.112 : +0.087	-0.113 : +0.084, t=-0.44,p=0.662

Supplementary table 2. Results of all statistical tests when Bayesian and Non-Bayesian regression models were used. Bold numbers indicate significant effects when the Credible intervals (CrI) or Confidence intervals (CI) do not overlap with 0. There were no discrepancies regarding significance of effects between Bayesian and Non-Bayesian methods. LFP, local field potential; RT, reaction time.

Statistical test (only correct trials)	CrI (Bayesian)	CI, t- & p-value (Frequentist)
<u>Reaction time:</u>		
Instruction	**-0.394 : -0.094**	**-0.382:-0.098, t=-3.31, p=0.003**
<u>Movement time:</u>		
Instruction	**-0.131 : -0.049**	**-0.129 : -0.050, t=-4.40,p<0.001**
Group	**-0.704 : -0.173**	**-0.704 : -0.197, t=-3.47,p=0.002**
<u>Single trial regression movement time~reaction time:</u>		
Movement time	**-0.128 : -0.027**	**-0.125 : -0.029, t=-3.14,p=0.003**
<u>Effect of LFP on movement time:</u>		
$Gamma_{move}$	**-0.058 : -0.003**	**-0.058 : -0.003, t=-2.16,p=0.031**
$Beta_{move}$*Instruction	**+0.004 : +0.080**	**+0.004 : +0.079, t=2.18,p=0.030**
$Beta_{move}$ (Speed)	**+0.010 : +0.070**	**+0.010 : +0.070, t=2.59,p=0.010**
<u>Effect of LFP on reaction time:</u>		
$Beta_{cue}$	**-0.135 : -0.041**	**-0.135 : -0.041, t=-3.64,p<0.001**
$Beta_{cue}$ (RT < 0.4 s excluded)	**-0.093 : -0.022**	**-0.092 : -0.022, t=-3.20,p=0.001**
$Theta_{cue}$*Instruction	**-0.138 : -0.007**	**-0.138 : -0.007, t=-2.18,p=0.030**

Supplementary table 3. Results from significant effects from supplementary table 2 when only correct trials are included. Bold numbers indicate significant effects. CrI, credible Interval; CI, confidence interval.

Statistical test (non-log-transformed)	CrI (Bayesian)	CI, t- & p-value (Frequentist)
Reaction time:		
Instruction	**-0.431 : -0.117**	**-0.425:-0.129, t=-3.65, p=0.001**
Movement time:		
Instruction	**-0.052 : -0.017**	**-0.051 : -0.017, t=-3.90,p<0.001**
Group	**-0.217 : -0.063**	**-0.215 : -0.071, t=-3.88,p<0.001**
Single trial regression movement time~reaction time:		
Movement time	**-0.109 : -0.012**	**-0.106 : -0.013, t=-2.51,p=0.017**
Effect of LFP on movement time:		
Gamma_{move}	**-0.025 : -0.006**	**-0.025 : -0.006, t=-3.34,p<0.001**
Beta_{move}*Instruction	**+0.001 : +0.025**	**+0.001 : +0.025, t=2.04,p=0.042**
Beta_{move} (Speed)	**+0.007 : +0.024**	**+0.007 : +0.024, t=3.51,p<0.001**
Effect of LFP on reaction time:		
Beta_{cue}	**-0.114 : -0.039**	**-0.114 : -0.039, t=-3.97,p<0.001**
Beta_{cue} (RT < 0.4 s excluded)	**-0.092 : -0.020**	**-0.092 : -0.021, t=-3.15,p=0.002**
Theta_{cue}*Instruction	-0.147 : + 0.001	**-0.147 : -0.001, t=-1.97,p=0.049**

Supplementary table 4. Results from significant effects from supplementary table 2 when dependent variables are not log-transformed. Bold numbers indicate significant effects. CrI, credible Interval; CI, confidence interval.

Baseline window:	-500 to -300 ms	-400 to -200 ms	-300 to -100 ms	-200 to 0 ms
reaction time:				
$Beta_{cue}$	**t=-3.43, p<0.001**	**t=-3.57, p<0.001**	**t=-3.46, p<0.001**	**t=-2.39, p=0.017**
$Beta_{cue}$ * Instruction	t=0.44, p=0.659	t=1.14, p=0.254	t=1.39, p=0.164	t=0.68, p=0.497
movement time:				
$Beta_{cue}$	t=0.17, p=0.865	t=0.47, p=0.640	t=0.58, p=0.561	t=1.06, p=0.288
$Beta_{cue}$ * Instruction	t=-1.04, p=0.300	t=-0.91, p=0.365	t=-1.03, p=0.303	t=-1.70, p=0.089

Supplementary table 5. Results from regression analyses (frequentist LME) between $Beta_{cue}$ and single trial behavioral data when using different baseline periods (ms in relation to cue onset) for extracting $Beta_{cue}$. Bold numbers indicate significant effects.

Inclusion of ramping in ON stimulation	20 ms ramping	40 ms ramping	60 ms ramping	80 ms ramping	100 ms ramping
Bilateral DBS	**t_9=-3.15, p=0.012**	**t_9=-3.10, p=0.013**	**t_9=-2.99, p=0.015**	**t_9=-2.83, p=0.020**	**t_9=-2.68, p=0.025**
Contralateral DBS	**t_7=-2.44, p=0.045**	**t_7=-2.52, p=0.040**	**t_7=-2.61, p=0.035**	**t_7=-2.73, p=0.029**	**t_7=-2.85, p=0.025**

Supplementary table 6. Results from DBS effects on reaction times when incremental parts of the ramping up and down period are defined as ON stimulation. Ramping in ms is the sum of ramping up and down, i.e. 100 ms corresponds to including the last 50 ms of ramping up and first 50 ms of ramping down. Note that when including 100 ms ramping (last column) only ~20% of trials were OFF stimulation trials. Results for bilateral DBS are computed from the DBS_{RT} window and for contralateral DBS from the DBS_{contra} window (see methods and main text). Bold numbers indicate significant effects.

Supplementary references:

1. Schade, S., Mollenhauer, B. & Trenkwalder, C. Levodopa Equivalent Dose Conversion Factors: An Updated Proposal Including Opicapone and Safinamide. *Mov Disord Clin Pract* **7**, 343-345 (2020).
2. Tomlinson, C.L.*, et al.* Systematic review of levodopa dose equivalency reporting in Parkinson's disease. *Mov Disord* **25**, 2649-2653 (2010).
