# Supplementary material for: Dynamic control of decision and movement speed in the human basal ganglia
Source: Nat Commun. 2022 Dec 7;13:7530. doi: 10.1038/s41467-022-35121-8 (PMC9729212; doi:10.1038/s41467-022-35121-8)
Supplement: Supplementary file 3 — Reporting Summary [file 41467_2022_35121_MOESM3_ESM.pdf]

nature portfolio

Corresponding author(s): Damian M. Herz

Last updated by author(s): 07.11.2022

Reporting Summary

Nature Portfolio wishes to improve the reproducibility of the work that we publish. This form provides structure for consistency and transparency in reporting. For further information on Nature Portfolio policies, see our Editorial Policies and the Editorial Policy Checklist.

Statistics

For all statistical analyses, confirm that the following items are present in the figure legend, table legend, main text, or Methods section.

n/a	Confirmed	
☐	☒	The exact sample size (*n*) for each experimental group/condition, given as a discrete number and unit of measurement
☐	☒	A statement on whether measurements were taken from distinct samples or whether the same sample was measured repeatedly
☐	☒	The statistical test(s) used AND whether they are one- or two-sided<br>*Only common tests should be described solely by name; describe more complex techniques in the Methods section.*
☐	☒	A description of all covariates tested
☐	☒	A description of any assumptions or corrections, such as tests of normality and adjustment for multiple comparisons
☐	☒	A full description of the statistical parameters including central tendency (e.g. means) or other basic estimates (e.g. regression coefficient) AND variation (e.g. standard deviation) or associated estimates of uncertainty (e.g. confidence intervals)
☐	☒	For null hypothesis testing, the test statistic (e.g. *F*, *t*, *r*) with confidence intervals, effect sizes, degrees of freedom and *P* value noted<br>*Give P values as exact values whenever suitable.*
☐	☒	For Bayesian analysis, information on the choice of priors and Markov chain Monte Carlo settings
☐	☒	For hierarchical and complex designs, identification of the appropriate level for tests and full reporting of outcomes
☐	☒	Estimates of effect sizes (e.g. Cohen's *d*, Pearson's *r*), indicating how they were calculated

Our web collection on statistics for biologists contains articles on many of the points above.

Software and code

Policy information about availability of computer code

Data collection

Data (neurophysiology and force) were collected on a TMSi porti device (TMS International, Enschede, The Netherlands) using TMSi software and drivers. PsychoPy v1.8 (https://www.psychopy.org/) was used for presenting stimuli and recording reaction times.

Data analysis

Statistical analyses were conducted using hierarchical Bayesian regression models (linear mixed models) implemented in R-Stan (v2.21.2, https://mc-stan.org) using the rethinking (v2.13, https://github.com/rmcelreath/rethinking) and rstanarm (v2.21.1, https://mc-stan.org/rstanarm) packages. For illustrations of the posteriors the bayesplot (v1.8.0, https://mc-stan.org/bayesplot) and ggplot2 (v3.4.0, https://ggplot2.tidyverse.org) packages were used. We also conducted the frequentist equivalents of these analyses with non-Bayesian linear mixed effect models and 95% confidence intervals (CI) using lme4 (v1.1.30, https://cran.r-project.org/web/packages/lme4/lme4.pdf) implemented in R (v4.0.5).
Drift Diffusion Modelling was conducted using a Bayesian hierarchical estimation of DDM (HDDM v0.8.0, http://ski.clps.brown.edu/hddm_docs/) implemented in Python3 (v3.6).
Neurophysiological data were analyzed in Matlab (R 2019a, The MathWorks, Natick, MA, USA) using FieldTrip (v20201126, https://www.fieldtriptoolbox.org/).
All code is made freely available on https://data.mrc.ox.ac.uk.

For manuscripts utilizing custom algorithms or software that are central to the research but not yet described in published literature, software must be made available to editors and reviewers. We strongly encourage code deposition in a community repository (e.g. GitHub). See the Nature Portfolio guidelines for submitting code & software for further information.

Data

Policy information about availability of data

All manuscripts must include a data availability statement. This statement should provide the following information, where applicable:

- Accession codes, unique identifiers, or web links for publicly available datasets
- A description of any restrictions on data availability
- For clinical datasets or third party data, please ensure that the statement adheres to our policy

Original data are available upon request to the corresponding author (damian.m.herz@gmail.com). At present, participant consent does not allow for depositing the full original dataset. A minimum example dataset (including scripts) is available on https://data.mrc.ox.ac.uk/data-set/subthalamic-nucleus-correlates-decision-and-movement-speed (doi: 10.5287/bodleian:1R9KzGXxM). Source data are provided with this paper.

Human research participants

Policy information about studies involving human research participants and Sex and Gender in Research.

Reporting on sex and gender

Participants of both biological sexes were included (13 male, 2 female in Parkinson group; 10 male, 5 female in healthy group). No subgroup analyses were performed.

Population characteristics

The Parkinson (PD) and healthy control (HC) groups were matched for age (PD: mean 67.4 years, range: 49-79; HC: mean 67.5 years, range: 57-81; P = 0.982, independent samples t-test), handedness (1 left handed person in each group as revealed by self-report, P = 1, Fischer's exact test) and gender (13 male in PD group, 10 male in HC group, P = 0.39, Fischer's exact test).

Recruitment

Thirteen patients with Parkinson's disease, who had undergone STN DBS surgery prior to the experimental recordings, and 15 people without any neurological or psychiatric conditions were recruited at the University Medical Center at the Johannes Gutenberg University Mainz, Germany.
Two patients with Parkinson's disease, who had undergone STN DBS surgery prior to the experimental recordings, were recruited at King's College Hospital London, UK.
The indication for DBS treatment were made purely on clinical grounds irrespective of the research study. Patients were asked if they were interested in participating in the study prior to surgery. Participation in the study did not affect the clinical treatment making self-selection bias less likely. To assess to what extent the enrolled patients were able to perform the task irrespective of their neurological condition they were compared to a group of healthy age-matched participants. These participants mainly consisted of people who had participated in previous unrelated studies, relatives or visitors, and did not receive payment for their participation.

Ethics oversight

The study was approved by the local ethics committees (State Medical Association of Rhineland-Palatinate and Oxfordshire REC A).

Note that full information on the approval of the study protocol must also be provided in the manuscript.

Field-specific reporting

Please select the one below that is the best fit for your research. If you are not sure, read the appropriate sections before making your selection.

☒ Life sciences ☐ Behavioural & social sciences ☐ Ecological, evolutionary & environmental sciences

For a reference copy of the document with all sections, see nature.com/documents/nr-reporting-summary-flat.pdf

Life sciences study design

All studies must disclose on these points even when the disclosure is negative.

Sample size

Before conducting the study we recorded pilot data in 12 healthy people performing the same experimental paradigm (described below). Our main focus was the effect of speed vs. accuracy (SAT) instructions on reaction and movement times. We used G*power to estimate the necessary sample size for a significant effect of SAT instructions on these two parameters and found an effect size of dz=2.3 for reaction times and 1.2 for movement times for a paired comparison. Given an alpha of 0.05 and power of 0.9 this resulted in a required sample size of n=10 for the lower effect size (movement times).
Due to the invasive nature of subthalamic nucleus (STN) recordings and deep brain stimulation (DBS) we were not able to record pilot data for computing the effect size of STN local field potential (LFP) changes or DBS effects. However, given the very good signal-to-noise ratio of invasive STN LFP recordings with a typical sample size of ~10 (see refs 16, 18, 20 in the article) and the large effect size of previous studies testing DBS effects during perceptual decision-making (dz ~1.8 and ~2.4, see refs 40 & 49 in the article) we considered the sample size estimation based on the behavioural measures appropriate. To also allow for possible drop-outs we opted to include 15 participants.

Data exclusions

All trials without responses (errors of omission), more than one response (i.e. if participants pressed the grippers twice in a trial) and response

Data exclusions	times < 0.25 s were excluded (see also ref 16 in the article).<br>Single trial LFP data with a z-score > 3 were excluded.
Replication	The study replicates several findings from previous studies (see article). The hierarchical Bayesian regression models and the Drift Diffusion modelling which formed the major analyses involved 3 Markov Chain Monte Carlo (MCMC) chains using 5000 and 10,000 iterations, respectively. When analysing critical periods in STN local field potentials we conducted control analyses shifting the baseline period across different time windows of the pre-cue period. When analysing the effects of burst stimulation we conducted additional control analyses defining incremental parts of ramping as stimulation and cluster-based permutation tests. Behavioral data was analyzed for all trials, only correct trials and non-logged transformed data. All results pertaining to Bayesian regression models, were replicated using the frequentist equivalent analyses (linear mixed effects models). Appropriate statistical methods and correction for multiple comparisons were used when appropriate. These different controls showed that our findings were robust with respect to within cohort manipulations.
Randomization	Since the study did not compare different treatments or manipulations for different groups, randomization was not applicable except from the third session (unilateral stimulation), in which the order of left vs. right STN stimulation was pseudo-randomized and counterbalanced. Of note, effects of stimulation were compared in a within-subject design and involved comparing time windows with vs. without burst stimulation in the same session (i.e. there was no order effect that could have been randomized).
Blinding	Since the study did not compare different treatments or manipulations for different groups, blinding was not applicable except from the third session (unilateral stimulation), in which patients (but not the researchers) were blinded to the side of stimulation. Of note, effects of stimulation were compared in a within-subject design and involved comparing time windows with vs. without burst stimulation in the same session (i.e. patients or researchers were not blinded to a stimulation or no-stimulation condition, but stimulation bursts, which could not be detected by the participants, occurred randomly during the stimulation sessions). For the manual detection of movement onset, the researcher was blinded to trial type (by-trial variations in Speed vs. Accuracy trials).

Reporting for specific materials, systems and methods

We require information from authors about some types of materials, experimental systems and methods used in many studies. Here, indicate whether each material, system or method listed is relevant to your study. If you are not sure if a list item applies to your research, read the appropriate section before selecting a response.

Materials & experimental systems

n/a	Involved in the study
☒	☐ Antibodies
☒	☐ Eukaryotic cell lines
☒	☐ Palaeontology and archaeology
☒	☐ Animals and other organisms
☒	☐ Clinical data
☒	☐ Dual use research of concern

Methods

n/a	Involved in the study
☒	☐ ChIP-seq
☒	☐ Flow cytometry
☒	☐ MRI-based neuroimaging
